# Current Concepts in Spinal Fusion: A Special Issue 

**DOI:** 10.1007/s11420-020-09757-5

**Published:** 2020-03-18

**Authors:** Sohrab Virk, Sheeraz Qureshi

**Affiliations:** Department of Orthopedic Surgery, Hospital for Special Surgery, 535 East 70th Street, New York, NY 10021 USA

The evolution of spinal fusion over the past 100 years has been dramatic, with changes reflected in the care provided to patients around the world. In this special issue of *HSS Journal*^®^, we present current practices to enhance spinal fusion among surgeons at the Hospital for Special Surgery (HSS) and other institutions. As guest editors, we have aimed to provide a snapshot of how top surgeons utilize fusion techniques and technologies in treating patients requiring spinal stability and/or deformity correction.

One theme of this issue is the research supporting treatments for challenging patient populations. Othman et al. systematically reviewed the literature on the use of minimally invasive spinal fusion techniques to treat obese patients (10.1007/s11420-019-09735-6). They found that minimally invasive techniques provide equivalent outcomes as open techniques, with lower rates of complications. This is an important consideration for a patient population that has traditionally been associated with high rates of complications after elective spinal surgery [3]. It is important to remember that obese patients still benefit from appropriately indicated surgeries [1], and Othman et al. have outlined minimally invasive techniques to help with these procedures. Multilevel anterior cervical discectomy and fusion (ACDF) has been associated with higher rates of pseudarthrosis [4]. In another article in this issue, McCarthy et al. review ways to augment multilevel ACDF with the use of biologics and graft selection (10.1007/s11420-019-09738-3). In addition, “adjacent segment disease” is a broad term that encompasses a number of difficult clinical problems including worsened stenosis, facet arthropathy, and disk degeneration. Louie et al. summarize a new manner for classifying the etiology of adjacent segment degeneration (10.1007/s11420-019-09723-w).

This special issue also outlines scientific innovations that have been adapted for use in spinal surgery. Greene et al. discuss strategies for taking developments in basic science to help solve difficult clinical questions in spinal fusion (10.1007/s11420-019-09733-8). This process might involve a “reverse translational pathway” to utilize clinical information and patient outcomes to guide basic science research. Developments in material science and nanotechnology have provided new materials that allow for osteointegration at the bone–implant interface during spinal fusion. Within our issue, Katsuura et al. outline new instrumentation that uses surface technology in order to increase osseous integration and enhance spinal fusion (10.1007/s11420-020-09752-w). Steinberger et al. discuss using all these innovations in interbody technology, osteobiologics, and instrumentation to further enhance spinal fusion for patients requiring surgery for spinal deformity (10.1007/s11420-020-09751-x).

We are proud to bring you this special issue, especially given the rich history of spinal surgery at HSS. HSS surgeon John Cobb, MD, was a giant in the field of treating patients with spinal deformity; his work included developing the “Cobb angle” to quantify a scoliotic curve and collaborating with other surgeons to refine the use of a turnbuckle cast, both of which contributed to the foundation of scoliosis correction in particular and spinal surgery in general [2]. Today, surgeons at HSS employ pre-operative planning, spinal instrumentation, osteobiologics, and interbody devices in seeking to augment healing for patients with spinal pathology. We have attempted to capture these advancements in this special issue of *HSS Journal*. The evolution of spinal fusion has been outlined by Virk et al. to provide context for the innovations discussed in other articles in our special issue (10.1007/s11420-020-09747-7).

We thank all authors for their contributions in gathering the data, the research, and the ideas required to produce an issue of this scope, summarizing the wide array of strategies available to surgeons in spinal fusion. We hope these articles will guide clinicians in better understanding the strategies for creating solid bony fusion for patients, and we encourage readers to use these articles in designing further research to improve outcomes associated with spinal surgery.

## Electronic supplementary material

ESM 1 (PDF 1225 kb)
